# Large Uterine Angiolipoleiomyoma Masquerading as an Ovarian Tumor: A Case Report

**DOI:** 10.1155/cris/8616290

**Published:** 2026-09-12

**Authors:** Prabineshwor Prasad Lekhak, Smriti Basnet, Hari Narayan Rai, Palistha Shakya, Tulsi Raj Khanal, Jyotsna Sharma Tiwari, Roshan Ghimire

**Affiliations:** ^1^ Department of General Surgery, Kathmandu Medical College Public Limited, Baburam Acharya Road Sinamangal, Kathmandu 44600, Nepal, kmc.edu.np; ^2^ Department of Obstetrics and Gynecology, Kathmandu Medical College Public Limited, Baburam Acharya Road Sinamangal, Kathmandu 44600, Nepal, kmc.edu.np; ^3^ Department of Radiology, Kathmandu Medical College Public Limited, Baburam Acharya Road Sinamangal, Kathmandu 44600, Nepal, kmc.edu.np

**Keywords:** angiolipoleiomyoma, large, uterus

## Abstract

Angiolipoleiomyoma (ALLM) is a rare benign mesenchymal tumor that primarily arises in the skin and kidney, and less frequently in the uterus. The present case may represent the largest reported uterine mass described as ALLM. We present a case of a 52‐year‐old woman with abdominal fullness and an irregular menstrual cycle having a large cystic abdominopelvic mass initially masquerading as a large ovarian tumor. Following successful tumor resection and subsequent histopathological and immunohistochemical analysis, a definitive diagnosis of uterine ALLM was established. This case shows that uterine ALLM can present with nonspecific clinical symptoms and radiologic features that mimic other pelvic pathologies, and should be considered in the differential diagnosis when evaluating pelvic masses in female patients.

## 1. Introduction

Angiolipoleiomyoma (ALLM) is a rare benign tumor composed of smooth muscle, mature adipocytes, and abnormal blood vessels, present in varying proportions, with a reported prevalence of only 0.06% among all benign uterine tumors [1]. Uterine ALLM occurs in both pre‐ and postmenopausal women [2]. Owing to its rarity, coupled with nonspecific or asymptomatic presentation mimicking other pelvic pathologies and the absence of distinct imaging features, preoperative diagnosis is a challenge [1, 3]. With fewer than 20 reported cases in the literature, definitive diagnosis relies on a multidisciplinary approach involving imaging, histopathological, and immunohistochemical analysis [4].

Reporting additional cases is essential to broaden the current understanding of this rare entity and its clinical and radiological spectrum, improve recognition, and address diagnostic challenges. This report describes a case of giant uterine ALLM in which accurate preoperative diagnosis was complicated by the massive size of the lesion and significant distortion of normal pelvic anatomy, which obscured the site of origin and mimicked an epithelial ovarian malignancy. This case strongly emphasizes the indispensable role of multimodal diagnostic correlation among radiological, intraoperative, histopathological, and immunohistochemical findings in establishing the correct diagnosis.

## 2. Case Presentation

A 52‐year‐old female (para 2, living 2) presented to the surgical outpatient department with progressive abdominal fullness, an irregular menstrual cycle for 1 year, and amenorrhea for the past 3 months. Her menstrual cycle had been irregular, occurring once every 2–3 months, with bleeding lasting 2–3 days, with no passage of clots or experiencing dysmenorrhea. Apart from hypertension for 1 year under medication, and a remote history of cholecystectomy (3 decades ago), she had no other significant medical, surgical, or gynaecological history, and no family history of malignancy. Her vital signs were stable at presentation. On per‐abdominal examination, a mass consistent with a 22–24‐week‐sized gravid uterus was palpable, with an increased abdominal girth. A vaginal examination revealed fullness in the bilateral fornixes.

**Figure 1:**
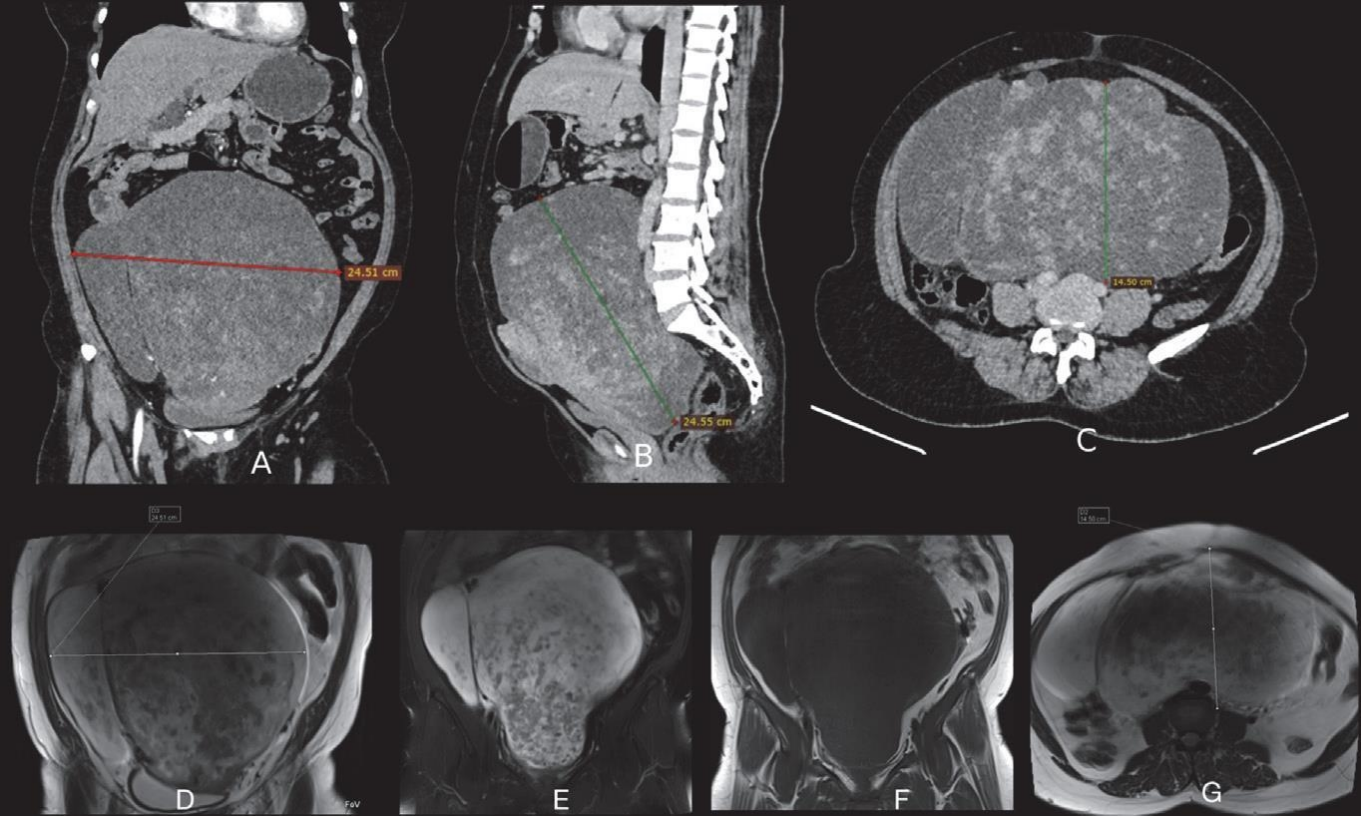
(A) Coronal view, (B) sagittal view, and (C) axial view of CECT of the abdomen and pelvis of the patient, showing well‐defined, large, heterogeneous, predominantly hypodense lesions with multiple soft‐tissue densities. (D) T2‐weighted coronal view, (E) T2‐weighted fat‐saturated image, (F) T1‐weighted coronal view, and (G) a T2‐weighted axial view of a noncontrast magnetic resonance study showing a mass with a predominantly cystic component.

Her complete hemogram and renal and hepatic function tests were normal. Blood beta‐human chorionic gonadotropin (β‐hCG) was within the non‐pregnant range. Ultrasonography (USG) of the abdomen and pelvis showed a large, heterogeneous, well‐vascularized, and irregularly echogenic abdominopelvic mass displacing the uterus. Next, contrast‐enhanced computed tomography (CECT) of the abdomen and pelvis was performed. A well‐defined, predominantly cystic lesion without calcification was noted in the abdominopelvic region, measuring 24.51 cm × 14.50 cm × 24.55 cm (Figure 1). Owing to the large size of the lesion, the exact origin of the lesion was difficult to establish. To ascertain the origin and characterization of the lesion, a complementary limited noncontrast magnetic resonance study was performed (Figure 1).

The lesion was supplied by a direct branch from the right internal iliac artery as well as a branch from the right uterine artery with venous drainage to the right ovarian vein. The right ovary was traced between the two loculations of this lesion, whereas the left ovary was displaced laterally. The uterus was elongated and distorted, measuring approximately 11.64 cm × 3.6 cm in size, with a normal endometrial lining. Bowel loops were displaced superiorly. No ascites were observed. This initially implied that the lesion was most likely a neoplastic right adnexal lesion. Subsequently, tumor markers were detected. The levels of blood cancer antigen‐125 (CA‐125), alpha‐feto protein (αFP), carcinoembryonic antigen (CEA), and cancer antigen‐19.9 (CA‐19.9) were all within normal limits. Because the imaging modality suggested a large cystic lesion with an apparent right adnexal origin, a right adnexal neoplasm was suspected. Given the imaging characteristics and indeterminate nature, the preoperative diagnosis included both benign and malignant ovarian epithelial neoplasms, including serous cystadenoma and serous cystadenocarcinoma. Although CA‐125, CEA, CA19‐9, and αFP were within normal limits, these findings do not reliably exclude ovarian malignancy. Therefore, preoperative diagnosis was primarily based on the imaging findings.

**Figure 2:**
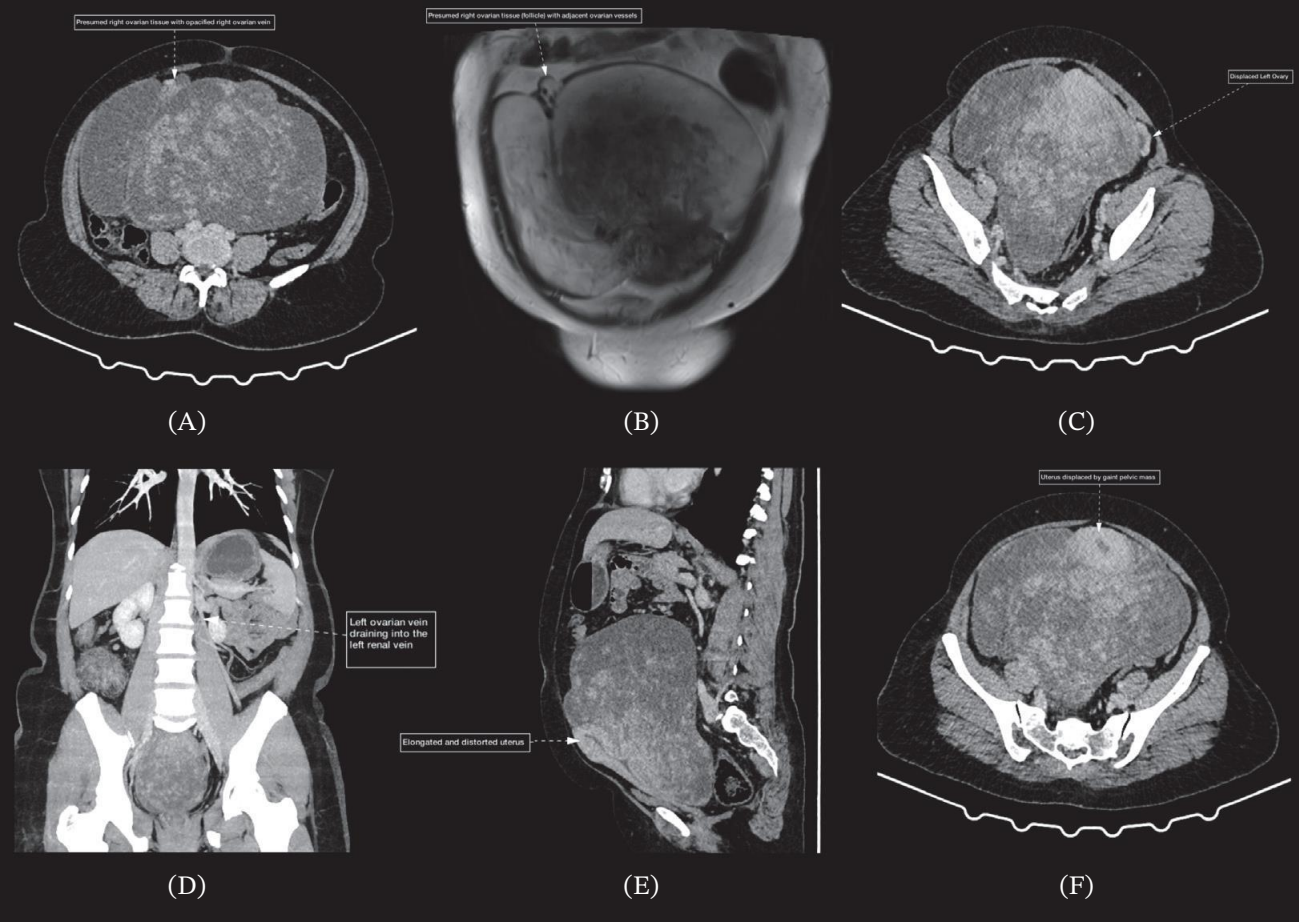
(A) Axial view of CECT of the abdomen and pelvis (venous phase) illustrating presumed right ovarian tissue (arrow) with an adjacent opacified right ovarian vein, favoring a preoperative diagnosis of a right ovarian mass. (B) Preoperative T2‐weighted coronal MRI demonstrating right ovarian tissue (arrow), identified by a follicular structure between two loculations of the mass and adjacent ovarian vessels. (C) Axial view of CECT of the abdomen and pelvis demonstrating the displaced left ovary (arrow), separately identified from the mass and visualized in relation to the left uterine cornu. (D) Maximum intensity projection (MIP) image from the coronal view of CECT (venous phase) of the abdomen and pelvis, depicting the left ovarian vein (arrow) traced from the left ovary to the left renal vein. (E) Sagittal view of CECT of the abdomen and pelvis demonstrating an elongated and distorted uterus (arrow). (F) Axial view of CECT of the abdomen and pelvis showing the uterus (arrow) displaced superiorly.

The giant pelvic mass caused significant distortion of the normal pelvic anatomy, limiting the accurate localization of its origin. The right ovary was not identified separately, while a follicular structure, interpreted as the right ovarian tissue (follicle), was situated between two loculations of the mass (Figure 2). Additionally, contrast‐enhanced CT showed venous drainage through the right ovarian vein, and the lesion demonstrated a predominantly cystic appearance on CT and magnetic resonance imaging (MRI), together suggesting a right ovarian epithelial neoplasm (Figure 2). In contrast, the displaced left ovary was identified and could be traced to the left uterine cornu (Figure 2). The uterus was clearly visualized and elongated and distorted, likely due to displacement by the large mass (Figure 2).

All routine preoperative investigations were sent (were within normal limits). A total abdominal hysterectomy along with bilateral salpingo‐oophorectomy (TAHBSO) and cystectomy was planned.

**Figure 3:**
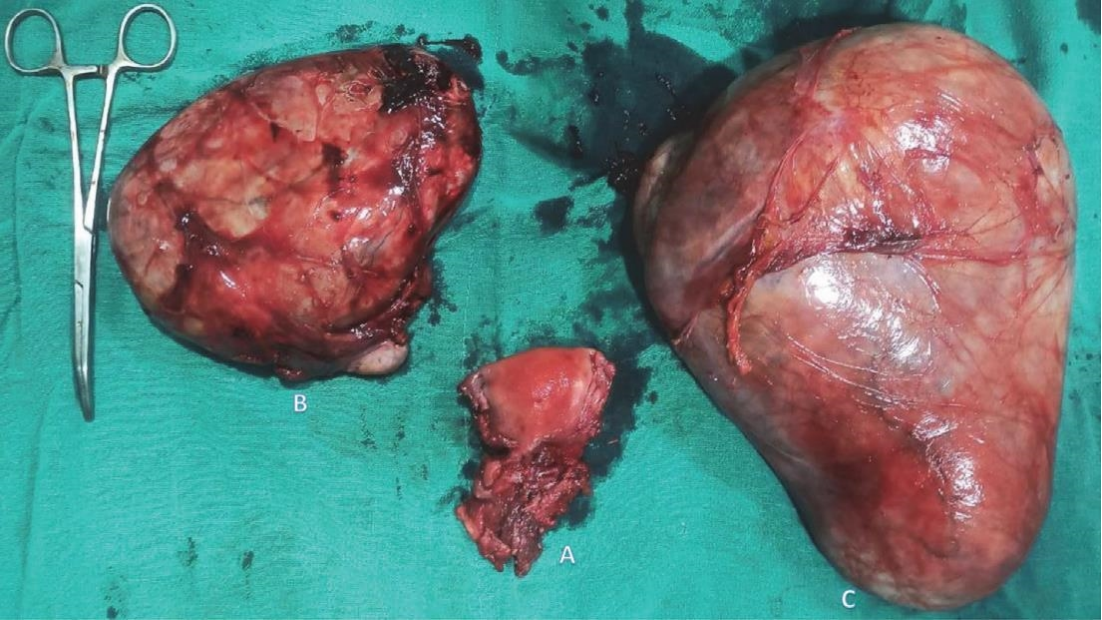
Photograph taken immediately after resection of the mass, displaying (A) a uterus and (B, C) a large abdominopelvic mass.

Under aseptic conditions, the abdomen was opened in layers. Upon gross examination, the uterus with the cervix was 10 cm × 5.5 cm × 1.2 cm in size. Contrary to expectations, intraoperatively, the bilateral tubes and ovaries appeared normal. The right ovary was found to be intact and markedly displaced. Two large masses with sizes of approximately 23.8 cm × 19.4 cm × 9.1 cm and 18.5 cm × 11.0 cm × 5.5 cm, both with smooth and glistening surfaces closely associated with the uterine corpus, were noted (Figure 3). A peritoneal wash was performed, and the sample was sent for cytology. A staged laparotomy was performed, and then, the mass was carefully excised along with the uterus, bilateral tubes, and ovaries. A Romovac drain was placed, after which the patient was closely monitored postoperatively.

The cut surface of the excised globular mass showed light yellow solid areas with multiple cystic cavities 0.2–3 cm in diameter. The cut section of the excised uterus revealed an empty uterine cavity.

**Figure 4:**
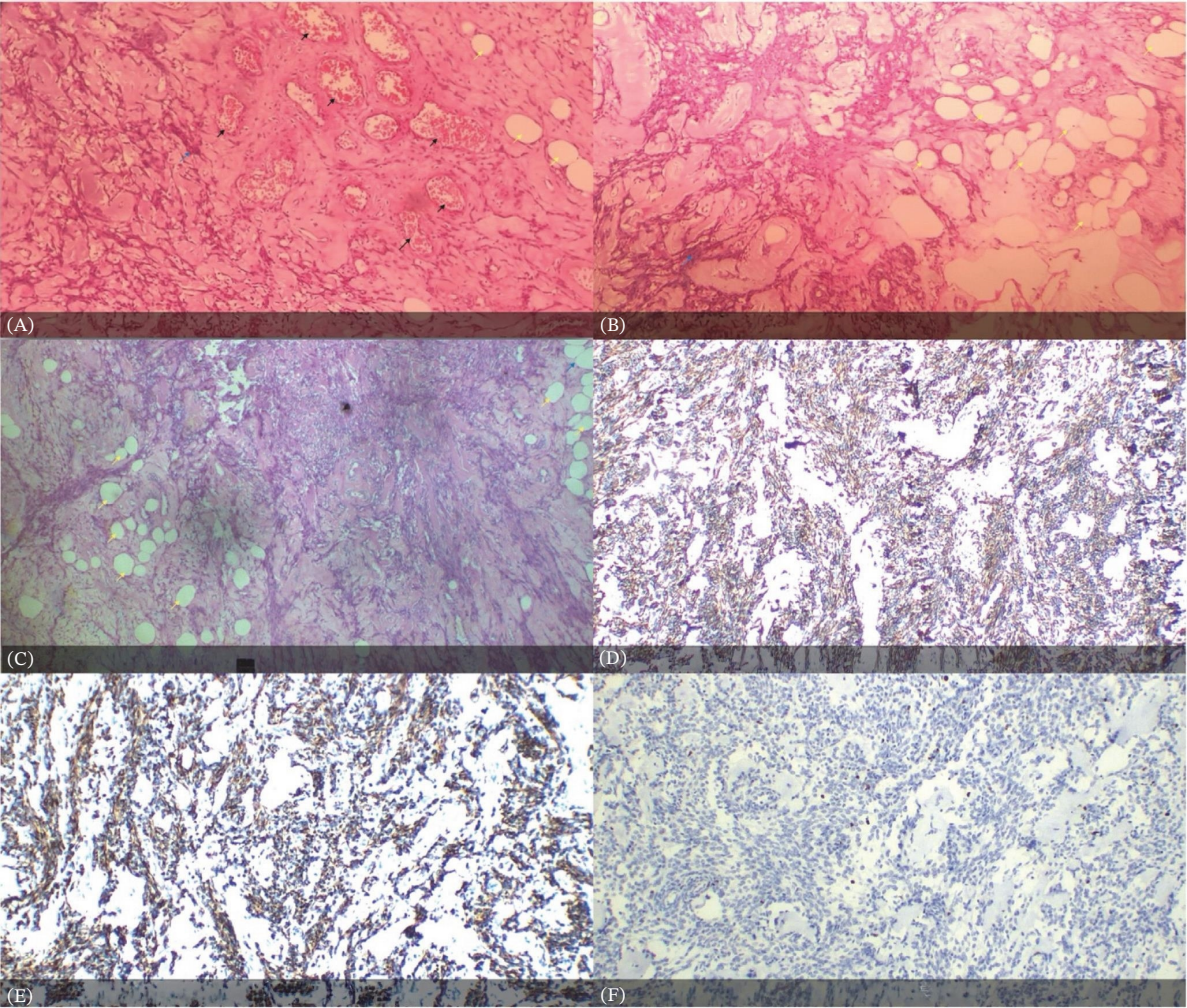
(A–C) show microscopic findings with hematoxylin and eosin (H&E) staining at 10x magnification from sections of both globular masses, which show well‐organized blood vessels congested with red blood cells (labeled with black arrows), aggregates of mature adipocytes containing clear cytoplasm, and peripherally located nuclei (labeled with yellow arrows), with spindle‐shaped smooth muscle cells (labeled with blue arrows). (D) Immunohistochemistry showing tumor cells positive for SMA at 10x magnification. (E) Immunohistochemistry showing tumor cells positive for Desmin at 10x magnification. (F) Immunohistochemistry showing Ki67 (~3%) in tumor cells at 10x magnification.

Microscopically, both globular masses showed well‐differentiated hypercellular areas with spindle‐ to stellate‐shaped tumor cells arranged diffusely, with focal aggregates of mature adipocytes and numerous blood vessels (Figure 4). No significant cytological atypia, coagulative tumor necrosis, or increased mitotic activity was noted. A customized immunohistochemistry (IHC) was performed. The tumor cells were positive for SMA and DESMIN and negative for HMB‐45, INHIBIN, calretinin, S‐100, CK, CD34, MUC4, and DOG1, with a low Ki‐67 proliferation index of approximately 3% (Figure 4). Based on combined morphologic and IHC results, a final diagnosis of uterine ALLM was established. Peritoneal fluid cytology was negative for malignant cells. The patient was then counseled on the benign nature of the disease.

The drain was removed on the 3^rd^ postoperative day, and the patient was discharged on the 5^th^ postoperative day. The patient was followed up for 12 months. She is currently doing well.

## 3. Discussion

ALLMs are found in organs such as the skin, kidney, and genital tract [5]. The uterine ALLM is an uncommon, benign mesenchymal tumor that is composed of blood vessels, smooth muscle, and adipocytes, with a prevalence rate of 0.06% among benign uterine lesions [1, 2, 4]. It is usually found in women in the fourth to sixth decade of life, with studies suggesting a reported mean age of presentation of 51.5 years [2, 4]. ALLM is usually asymptomatic but can display features of abdominopelvic pain, abdominal fullness, menstrual cycle disturbances—such as menometrorrhagia and postmenopausal bleeding, and pressure symptoms such as urinary frequency and prolapse [2, 4].

A pelvic mass in a female individual may be due to gynecological causes—such as ovarian (endometriosis, cysts, benign, or malignant cancers), fallopian (pelvic inflammatory disease, hydrosalpinx, and para‐ovarian cyst), or uterine (neoplasm, fibroma, and pyometra)—or nongynecological causes, such as gastrointestinal (neoplasms, abscesses, and impactions), urinary (bladder tumors and urachal cysts) and other miscellaneous conditions (peritoneal carcinomatosis, musculo‐skeletal tumors, pelvic vessel aneurysms, foreign bodies, and hematomas) [6]. Considering the variety of symptoms associated with uterine ALLM, it is often difficult to differentiate it clinically from these pathological conditions. USG is typically the first diagnostic step [6]. However, in cases where it is difficult to evaluate these lesions, CT and MRI are better at detecting the exact origin and composition of these lesions [6].

A PubMed search along with a Google Scholar search was performed up to 18 February 2025 using the keywords “uterine angiolipoleiomyomas” and “ALLM.” Eighteen cases of human uterine ALLM, written in English, were found and are summarized in Table 1.

**Table 1:** Worldwide case reports describing uterine angiolipoleiomyoma in humans.

S.N.	References/DOI	Country	Year	Age (years)	Location	Size (cm) (approximate)	Treatment	Was cystic component present?
1	Gupta et al. [7]	India	2024	40	Corpus	22.0 × 23.7 × 11.4	Total abdominal hysterectomy and right salpingo‐oophorectomy	Yes
2	Jayalakshmy et al. [5]	India	2024	54	Corpus	2.5 × 2 × 2 and 3 × 2.5 × 1.0	TAHBSO	Yes
3	Psomiadou et al. [4]	Greece	2022	59	Corpus	5.5 × 2.5	Total abdominal hysterectomy	Yes
4	Seo et al. [3]	South Korea	2022	50	Corpus	NA	Total abdominal hysterectomy	No
5	Verocq et al. [8]	Belgium	2022	58	Corpus	4.0 × 3.0 × 3.0	Total hysterectomy with bilateral Adnexectomy	Yes
6	Garg and Tanveer [9]	India	2021	46	Corpus	6.0 × 4.2 × 2.8	TAHBSO	No
7	Sulkar and Swami [2]	India	2021	41	Corpus	8.0 × 8.0 × 5.0	TAHBSO	Yes
8	Paryani and Shahid [10]	Pakistan	2020	26	Corpus	12.0 × 10.0 × 8.0	Myomectomy and plication of the round ligament (to preserve fertility)	No
9	Demir Cendek et al. [1]	Turkey	2018	69	Corpus	6.0 × 6.0	TAHBSO	No
10	Shakarwal et al. [11]	India	2017	28	Broad ligament	9.0 × 9.0	Enucleated and removed	Yes
11	Kajo et al. [12]	Slovakia	2010	53	Corpus	6.0 cm and 2.0 cm diameter each	Simple abdominal hysterectomy	No
12	Ren and Wu [13]	Germany	2004	40	Corpus	5.0 × 4.5 × 4.0	Hysterectomy	Yes
13	Braun et al. [14]	USA	2002	51	Corpus	2.3 × 1.3 × 1.3	TAHBSO	No
14	Shintaku [15]	Japan	1996	67	Corpus	7.0 cm diameter	NA	NA
15	Sieiński [16]	Poland	1989	52	Corpus	6.0 cm diameter	Hysterectomy	NA
16				52	Cervix	16.0 cm diameter	Hysterectomy	NA
17				57	Cervix	9.0 cm diameter	Hysterectomy	NA
18	Lo Re et al. [17]	Italy	1987	47	Corpus	4.0 cm diameter	NA	NA

*Note:* NA, data not recorded or data not found.

The uterine ALLM has no established characteristic imaging findings [3]. However, imaging modalities such as USG, CT, and MRI are useful in the diagnosis [2]. On USG, a sharply demarcated, well‐vascularized mass with increased echogenicity is typically observed in uterine ALLM [14]. In this case, the heterogeneity and irregularly echogenic areas may have been due to the large size of the mass, which likely resulted in increased cystic degeneration or necrosis. The uterine ALLM can have variable appearances on CT scans, as one of its three components may be more prominent than the others [10]. A CT scan may show a heterogeneous pelvic mass with variable fat and soft‐tissue components, while an MRI scan has a better chance of characterizing the lesion as a heterogeneous uterine mass with inhomogeneous signal intensity and enhancement; however, these findings are nonspecific and cannot be generalized [3, 4]. Hence, histopathological analysis is considered a cornerstone for definitive diagnosis [3, 4]. The possible differentials of the imaging findings include lipo‐leiomyoma, leiomyoma with degenerative changes, mature cystic teratoma, fat‐containing ovarian neoplasms, and angiomyolipoma (AML) [4, 6]. Mature cystic teratoma has imaging features such as calcifications, teeth, hair, and sebaceous components [6]. Lipo‐leiomyoma lacks a blood vessel component histologically, in contrast to ALLM having thick‐walled blood vessels [4, 15, 17]. AML can be distinguished according to its immunoreactivity for melanocytic markers, which shall be discussed later in this study.

The blood supply to the mass suggested right‐sided pathology. The lack of evidence to separately identify the right ovary, together with venous drainage by the ovarian vein and cystic morphology of the lesion, reasonably implicated an ovarian origin, likely an epithelial neoplasm. However, on surgical exploration, the presumed ovarian tissue between two loculations represented the intact, markedly displaced right ovary, and the mass was confirmed to be of uterine origin. On retrospective review, the marked elongation and distortion of the uterus could be pointed out as a potential clue to its uterine origin. Nevertheless, given the enormous size of the tumor and the resultant distortion of normal pelvic anatomy, it was not possible to definitively determine preoperatively whether these uterine changes represented the site of origin or secondary displacement by the mass. Therefore, in a massive pelvic tumor with marked distortion of the uterus, adnexa, and pelvic vasculature, it may be difficult to determine the true site of origin, posing a great challenge to accurate preoperative localization despite multimodality imaging. This case is consistent with a previously reported case in which a large uterine tumor had a masquerading effect as another ovarian tumor [18]. Similar diagnostic pitfalls have been described recently by Hoang et al. [19] as well, where giant uterine leiomyomas with cystic or hydropic degeneration closely mimicked an ovarian neoplasm. Both benign and malignant ovarian epithelial lesions were considered in the differential diagnosis of the mass initially, despite normal CA‐125, CEA, CA19‐9, and FP levels because, as suggested by the literature and National Institute for Health and Clinical Excellence (NICE) guidelines, it should never be used to exclude ovarian cancer [20].

In the review of 18 cases, the ALLM arose from the body of the uterus in 15 cases (83.33%), similar to our case. The reported maximum length of the uterine ALLM ranged from 2 to 23.7 cm, with a median size of 6 cm. Notably, our case represents the largest reported uterine ALLM recorded to date, both radiologically and intraoperatively, as the CECT was 24.51 cm × 14.50 cm × 24.55 cm, and two masses were measured: 23.8 cm × 19.4 cm × 9.1 cm and 18.5 cm × 11.0 cm × 5.5 cm. In uterine ALLM, hysterectomy is the preferred method of treatment, along with resection of the mass [1]. Only one (5.55%) case in which a myomectomy was performed to save fertility has been reported in the literature.

Macroscopically, ALLM presents as a firm, nodular, encapsulated mass with white, gray, yellowish, or pinkish areas visible on the cut section. Based on the proportion of its contents, it can be a solid or soft mass. A cystic component was observed in seven (38.88%) out of 18 cases, which is consistent with our findings.

Microscopically, uterine ALLM contains three components: blood vessels, smooth muscle, and mature adipocytes [4]. Similarly, AML also contains the same components. Although these two entities differ in histology, clinical presentation, and terminology, the term ALLM has been used interchangeably with AML, a leiomyoma variant of AML, or smooth muscle‐predominant AML in the literature [9, 21–23].

AMLs, which are usually found in the kidney, are typically associated with the tuberous sclerosis complex (TSC), whereas ALLMs are not. Compared with AML, ALLM is characterized histologically by well‐differentiated, organized smooth muscle components that resemble leiomyoma without atypical features or mitotic activity and contain mature adipocytes [9, 12]. IHC plays a crucial role in differentiating these tumors. AMLs are generally HMB‐45 positive and weakly DESMIN positive, whereas ALLMs are generally HMB‐45 negative and strongly DESMIN positive [22]. However, extrarenal AML, which lacks an epithelioid component, can also be HMB‐45 negative and may not be linked to TSC [9]. Consequently, some authors propose that HMB‐45‐negative AMLs without a history of TSC, when located close to the uterus, should be termed ALLMs [12, 24].

In our patient, there were no clinical signs or symptoms suggestive of TSC. Histopathological examination also revealed well‐distinguished smooth muscle cells along with mature adipocytes. IHC revealed negative HMB‐45 staining and strong DESMIN positivity, from which we concluded that it was a uterine ALLM. Thus, uterine ALLM is a rare condition that warrants consideration when evaluating the pelvic masses in females. Imaging modalities such as CT and MRI in conjunction with immunohistochemical and histopathological examination can lead to a successful diagnosis even in the presence of clinical or radiological overlap with other pelvic pathologies.

**Nomenclature**
β‐HCG: Human chorionic gonadotropin
αFP: Alpha‐feto protein
ALLM: Angiolipoleiomyoma
AML: Angiomyolipoma
CA‐125: Cancer antigen‐125
CA‐19.9: Cancer antigen‐19.9
CEA: Carcinoembryonic antigen
CECT: Contrast‐enhanced computed tomography
CT: Computed tomography
H&E: Hematoxylin and eosin
MRI: Magnetic resonance imaging
NA: Data not recorded or data not found
TAHBSO: Total abdominal hysterectomy with bilateral salpingo‐oophorectomy
TSC: Tuberous sclerosis complex
USG: Ultrasonography.

## Funding

No funding was provided for the study.

## Ethics Statement

Ethical approval was not required for this anonymized case report in compliance with the institutional policy.

## Consent

A comprehensive, well‐written consent was obtained from the patient for the use of case information and accompanying images in this case report. All identifying information of the patient has been anonymized to protect patient confidentiality. The patient has agreed to the publication of these materials.

## Conflicts of Interest

The authors declare no conflicts of interest.

## Data Availability

The data that support the findings of this study are available from the corresponding author upon reasonable request.
